# Supplementary material for: Immune-deficient bacteria serve as gateways to genetic exchange and microbial evolution
Source: Nat Commun. 2026 Apr 1;17:4737. doi: 10.1038/s41467-026-71467-z (PMC13216270; doi:10.1038/s41467-026-71467-z)
Supplement: Supplementary file 1 — Supplementary Information [file 41467_2026_71467_MOESM1_ESM.pdf]

Supplementary Material

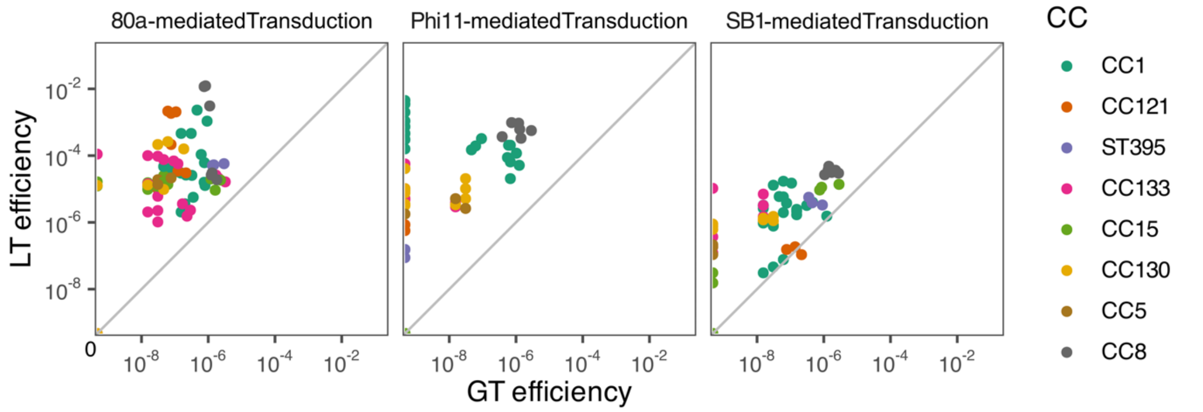

Figure S1. Comparison of transfer efficiency of chromosomal markers by lateral and generalised transduction. Three chromosomal markers were transferred from a CC8 donor by either lateral (y-axis) or generalised transduction (x-axis): first marker was mobilised by phage 80a, second marker by phage Phi11 and third marker by PICI SaPIbov1. Dots on the diagonal line indicate the transfer efficiency is the same by either mechanism (n=3 biological replicates). Dots above the line indicate that the efficiency is higher by lateral transduction.

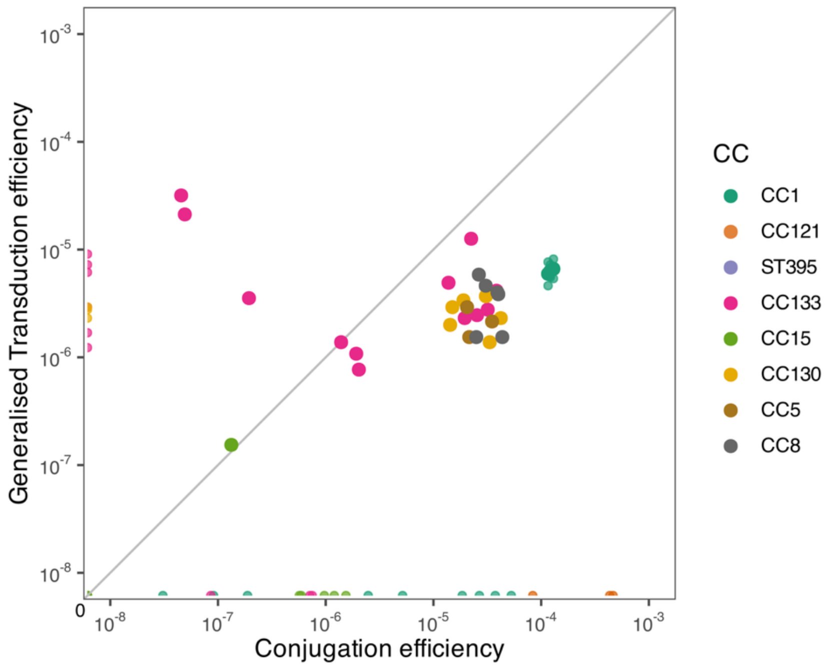

Figure S2. Comparison of plasmid transfer by transduction and conjugation. Plasmid pC221 was mobilised from a CC8 donor by either 80a-mediated generalised transduction (y-axis) or pGO1-mediated conjugation (x-axis) (n=3 biological replicates). Dots above the diagonal line indicate that the transfer efficiency is higher by generalised transduction, whereas dots below the line indicate that the efficiency is higher by conjugation.

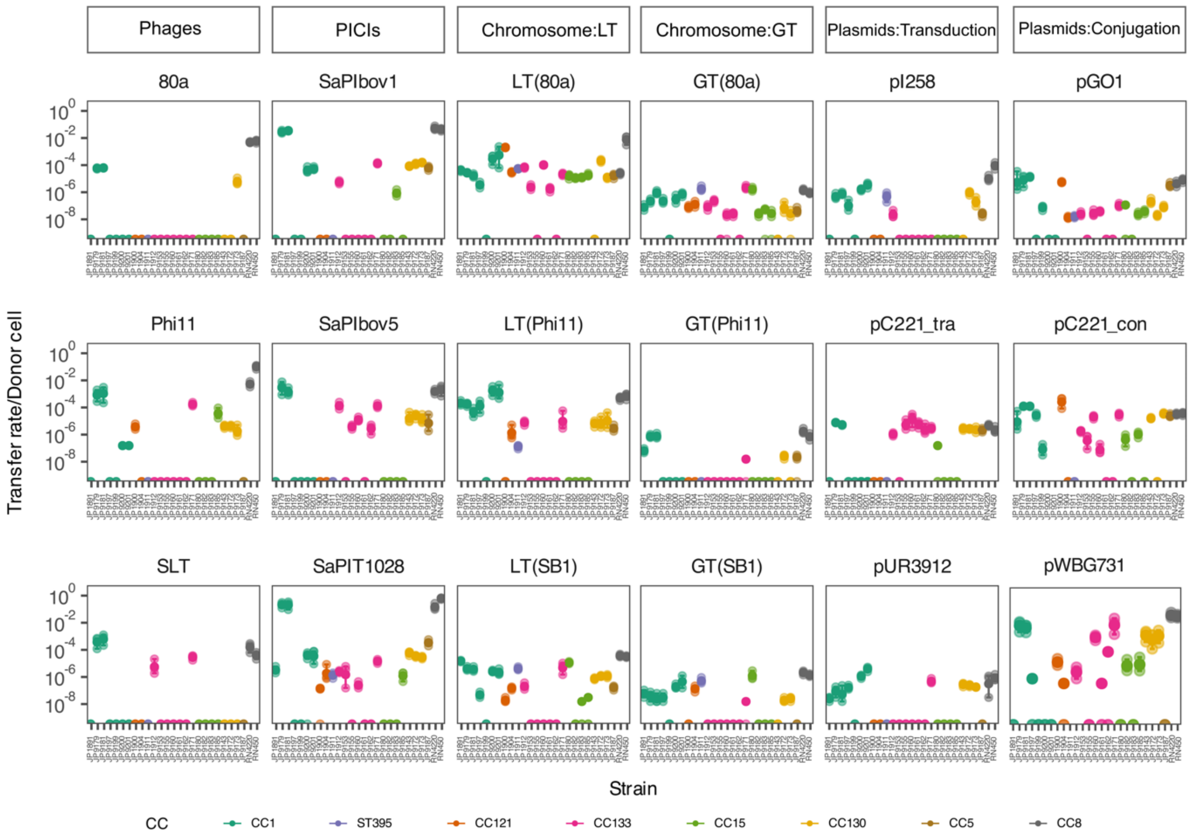

Figure S3. MGEs and chromosomal markers' mobility. The transfer efficiency of all the MGEs and chromosomal markers that were assessed in this study is shown in the figure n=3 biological replicates). The legend at the top of each column indicates the type of MGE (*e.g.,* first column shows phages, whereas the second column shows PICIs).

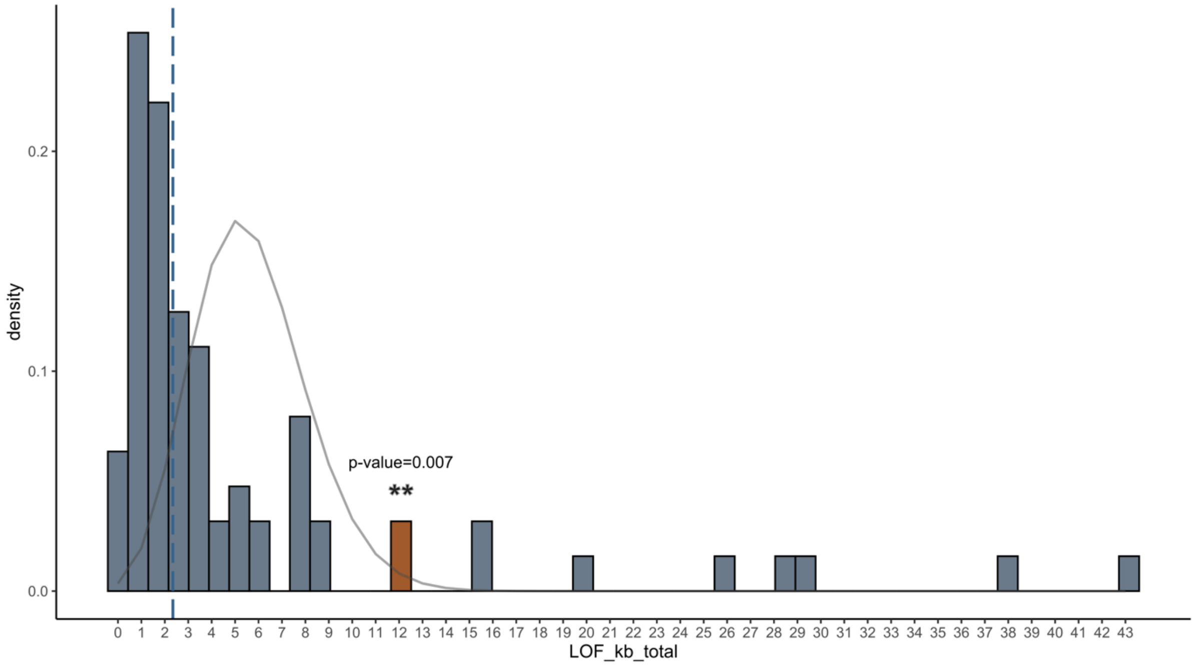

Figure S4. Density plot of medium genes with loss-of-function mutations. The total number of LOF mutations found in the complete *S. aureus* genomes was normalised to the gene size in kb. Only medium-sized genes (2-3kb) were included, and a Poisson distribution was calculated (grey curve). Bars above the Poisson distribution line indicate a frequency higher than expected by chance. The brown bar represents the mutations in *hsdR* (p-value=0.007 by Poisson test).

Table S1. Bacterial isolates information.

Strain	Source	Host	ST	CC	Reference
JP1891	Laboratory Centre for Disease Control (Ottawa): 3A	Unknown	ST9	CC1	[1]
JP9179	Copenhagen Zoo	Banded Mongoo	ST1	CC1	[2]
JP9181	Copenhagen Zoo	Banded Mongoo	ST1	CC1	[2]
JP9197	Copenhagen Zoo	Chimpanzee	ST188	CC1	[2]
JP9199	Copenhagen Zoo	Chimpanzee	ST188	CC1	[2]
JP9200	Copenhagen Zoo	-	ST188	CC1	[2]
JP9201	Copenhagen Zoo	-	ST188	CC1	[2]
JP1900	Laboratory Centre for Disease Control (Ottawa): 55	Unknown	ST95	CC121	[1]
JP1904	Laboratory Centre for Disease Control (Ottawa): 71	Unknown	ST95	CC121	[1]
JP1911	Felix d'Hérelle Reference Center for Bacterial Viruses: 187	Unknown	ST394	ST395	[1]
JP1912	National Collection of Type Cultures: X2	Unknown	ST133	ST133	[1]
JP9153	Copenhagen Zoo	Capybara	ST133	ST133	[2]
JP9155	Copenhagen Zoo	Goat	ST133	ST133	[2]
JP9160	Copenhagen Zoo	Goat	ST133	ST133	[2]
JP9161	Copenhagen Zoo	Goat	ST133	ST133	[2]
JP9162	Copenhagen Zoo	Goat	ST133	ST133	[2]
JP9171	Copenhagen Zoo	Malayan Tapir	ST133	ST133	[2]
JP9180	Copenhagen Zoo	Banded Mongoo	ST15	CC15	[2]
JP9182	Copenhagen Zoo	Egyptian fruitbat	ST15	CC15	[2]
JP9183	Copenhagen Zoo	Egyptian fruitbat	ST15	CC15	[2]
JP9185	Copenhagen Zoo	Egyptian fruitbat	ST15	CC15	[2]
JP9143	Copenhagen Zoo	Mara	ST130	ST130	[2]
JP9172	Copenhagen Zoo	Mara	ST130	ST130	[2]
JP9173	Copenhagen Zoo	Mara	ST130	ST130	[2]

JP9187	Copenhagen Zoo	Goat	ST6	CC5	[2]
RN4220	Reference	Laboratory	ST8	CC8	[3]
RN450	Reference	Laboratory	ST8	CC8	[3]

Table S2. Mobile Genetic Elements information.

MGE	Type	Description	Reference
80α	Phage	*S. aureu*s phage, 43.8 kb; aids mobilisation of a variety of PICIs. It carries immune systems Tha and AbiAlpha.	[3]
φSLT	Phage	*S. aureu*s phage, 42.9 kb; encodes Panton-Valentine leukocidin (PVL) virulence factors genes lukS and lukF.	[4]
φ11	Phage	*S. aureu*s phage, 43.6 kb. It carries immune system Tha.	[3]
SaPIbov1	PICI	*S. aureu*s PICI, 15.8 kb; encodes toxins *tst*, *sec*-bovine, and a *sel.*	[5]
SaPIbov5	PICI	*S. aureu*s PICI, 13.5 kb; encodes Willebrand factor-binding protein (vWb).	[6]
SaPIT1028	PICI	*S. aureu*s PICI, 15.6 kb; encodes anti-phage genes. It carries immune system sma.	[7]
pI258	Plasmid	*S. aureus* plasmid, 29 kb; confers resistance to erythromycin, cadmium, arsenic, and mercury.	[8]
pC221	Plasmid	*S. aureus* mobilizable plasmid, 4.6 kb; confers resistance to chloramphenicol.	[9]
pUR3912	Plasmid	*S. aureus* plasmid, 6.1 kb; confers resistance to erythromycin and cadmium.	[10]
pGO1	Plasmid	*S. aureus* conjugative plasmid, 54 kb; confers resistance to bleomycin, gentamicin, kanamycin, neomycin, quaternary ammonium compounds, and trimethoprim. It carries anti-CRISPR acriia21.	[11]
pWBG731	Plasmid	*S. aureus* conjugative plasmid, 74kb conjugative plasmid containing *bla* and *cadA* genes, acknowledgment for Joshua Ramsay, Curtin University)	[12]

Table S3. Mobile Genetic Element content of strains in the collection.

Strain	ST	CC	Phage Integrases*	PICI Integrases**	Plasmid PLSDB hits^
JP9179	ST1	CC1	None	SaPI V	1
JP9181	ST1	CC1	None	SaPI V	1
JP1891	ST9	CC1	Sa3	None	None
JP9199	ST188	CC1	Sa1,Sa2,Sa3,Sa4,Sa6	None	4
JP9197	ST188	CC1	Sa3,Sa9	None	3
JP1900	ST95	CC121	Sa2,Sa3,Sa4,Sa6	SaPI V	None
JP1904	ST95	CC121	Sa2,Sa3,Sa4,Sa6	SaPI V	None
JP9143	ST130	CC130	Sa3,Sa8	None	None
JP9172	ST130	CC130	Sa3,Sa8	None	None
JP9173	ST130	CC130	Sa3,Sa8	None	None
JP1912	ST133	CC133	Sa2,Sa4,Sa5,Sa6,Sa9	SaPI II, SaPI V	None
JP9160	ST133	CC133	Sa2,Sa4,Sa5,Sa6,Sa7,Sa9	SaPI V	None
JP9161	ST133	CC133	Sa2,Sa4,Sa5,Sa6,Sa7,Sa9	SaPI V	None
JP9162	ST133	CC133	Sa2,Sa4,Sa5,Sa6,Sa7,Sa9	SaPI V	None
JP9153	ST133	CC133	Sa2,Sa4,Sa6,Sa7,Sa8,Sa9	SaPI I, SaPI II	None
JP9155	ST133	CC133	Sa2,Sa4,Sa5,Sa6,Sa7,Sa9	SaPI V	None
JP9171	ST133	CC133	Sa2,Sa4,Sa6,Sa8,Sa9	SaPI I, SaPI II, SaPI V	None
JP9182	ST15	CC15	None	SaPI I	None
JP9183	ST15	CC15	None	SaPI I	None
JP9185	ST15	CC15	Sa2,Sa4,Sa6	SaPI I	None
JP9180	ST15	CC15	Sa2,Sa3,Sa4,Sa6	None	None
JP1911	ST395	ST395	Sa2,Sa3,Sa4,Sa6	SaPI IV, SaPI V	1
JP9187	ST6	CC5	None	None	1
RN4220	ST8	CC8	None	None	None
RN450	ST8	CC8	None	None	None

*Presence of phage integrases based on protein Blast. **Presence of SaPI integrases based on protein Blast. ^Number of plasmids identified based on nucleotide Blast against the plasmid database PLSDB.

Table S4. DNA motif recognised by target recognition domain of T1RM systems in the collection.

Strain	ST	CC	Type_I_RM-MS1*	Type_I_RM-MS2**
CH9179	ST1	CC1	CCAYNNNNNTTAA	CCAYNNNNNNTGT
CH9181	ST1	CC1	CCAYNNNNNTTAA	CCAYNNNNNNTGT
CH9200	ST188	CC1	unknown	unknown
CH9199	ST188	CC1	CCAYNNNNNTTAA	GGANNNNNNNTGC
CH9197	ST188	CC1	unknown	GGANNNNNNNTGC
CH1891	ST9	CC1	TCTANNNNNNTTAA	GAAGNNNNNNTTRG
CH1900	ST95	CC121	GGANNNNNNCCT	GACNNNNNNTAYG
CH1904	ST95	CC121	GGANNNNNNCCT	GACNNNNNNTAYG

CH9183	ST15	CC15	unknown	unknown
CH9185	ST15	CC15	unknown	unknown
CH9180	ST15	CC15	unknown	unknown
CH9182	ST15	CC15	unknown	unknown
CH9187	ST6	CC5	GGANNNNNNGTA	CCAYNNNNNNTGT
CH9143	ST130	ST130	CAAGNNNNNCTT	CCAYNNNNNNTGT
CH9172	ST130	ST130	CAAGNNNNNCTT	CCAYNNNNNNTGT
CH9173	ST130	ST130	CAAGNNNNNCTT	CCAYNNNNNNTGT
CH1912	ST133	ST133	CAGNNNNNRTGA	GGANNNNNNNTTRG
CH9160	ST133	ST133	CAGNNNNNRTGA	unknown
CH9161	ST133	ST133	CAGNNNNNRTGA	unknown
CH9162	ST133	ST133	CAGNNNNNRTGA	unknown
CH9153	ST133	ST133	unknown	GGANNNNNNNTTRG
CH9155	ST133	ST133	CAGNNNNNRTGA	unknown
CH9171	ST133	ST133	CAGNNNNNRTGA	GGANNNNNNNTTRG
CH1911	ST395	ST395	unknown	GAGNNNNNNTCG
RN4220	ST8	CC8	AGGNNNNNGAT	CCAYNNNNNNTGT
RN450	ST8	CC8	AGGNNNNNGAT	CCAYNNNNNNTGT

*DNA motif recognised by the specificity subunit located in genomic island vSaα. **DNA motif recognised by the specificity subunit located in genomic island vSaβ.

Table S5. Core SNP distance matrix of CC1 strains.

Strain	ST	JP1891	JP9179	JP9181	JP9197	JP9199	JP9200	JP9201
**JP1891**	ST9	0	42112	42290	50407	50346	51050	50683
**JP9179**	ST1	42112	0	214	35694	35622	36435	35966
**JP9181**	ST1	42290	214	0	35506	35434	36247	35778
**JP9197**	ST188	50407	35694	35506	0	1068	851	310
**JP9199**	ST188	50346	35622	35434	1068	0	1703	1234
**JP9200**	ST188	51050	36435	36247	851	1703	0	547
**JP9201**	ST188	50683	35966	35778	310	1234	547	0

Table S6. Closest relatives among CC1 strains.

Strain	Closest to	Distance (Core SNPs)
JP1891	JP9179	42112
JP9179	JP9181	214
JP9181	JP9179	214
JP9197	JP9201	310
JP9199	JP9197	1068
JP9200	JP9201	547
JP9201	JP9197	310

References

1. Kwan, T. et al. The complete genomes and proteomes of 27 Staphylococcus aureus bacteriophages, *Proc. Natl. Acad. Sci. U.S.A.* **102** (14) 5174-5179 (2005).
2. Espinosa-Gongora, C. et al. Occurrence and distribution of Staphylococcus aureus lineages among zoo animals. *Veterinary Microbiology* **158**, 228-231 (2012).
3. Novick, R. Properties of a cryptic high-frequency transducing phage in Staphylococcus aureus. *Virology* **33**, 155–166 (1967).
4. Narita, S. *et al.* Phage conversion of Panton-Valentine leukocidin in Staphylococcus aureus: molecular analysis of a PVL-converting phage, φSLT. *Gene* **268**, 195–206 (2001).
5. Fitzgerald, J. R. *et al.* Characterization of a Putative Pathogenicity Island from Bovine Staphylococcus aureus Encoding Multiple Superantigens. *Journal of Bacteriology* (2001) doi:10.1128/jb.183.1.63-70.2001.
6. Viana, D. *et al.* Adaptation of Staphylococcus aureus to ruminant and equine hosts involves SaPI-carried variants of von Willebrand factor-binding protein. *Molecular Microbiology* **77**, 1583–1594 (2010).
7. Kwan, T., Liu, J., DuBow, M., Gros, P. & Pelletier, J. The complete genomes and proteomes of 27 Staphylococcus aureus bacteriophages. *Proceedings of the National Academy of Sciences* **102**, 5174–5179 (2005).
8. Laddaga, R. A., Chu, L., Misra, T. K. & Silver, S. Nucleotide sequence and expression of the mercurial-resistance operon from Staphylococcus aureus plasmid pI258. *Proc Natl Acad Sci U S A* **84**, 5106–5110 (1987).
9. Projan, S. J. *et al.* Comparative sequence and functional analysis of pT181 and pC221, cognate plasmid replicons from Staphylococcus aureus. *Mol Gen Genet* **199**, 452–464 (1985).
10. Gómez-Sanz, E. *et al.* Analysis of a novel erm(T)- and cadDX-carrying plasmid from methicillin-susceptible Staphylococcus aureus ST398-t571 of human origin. *J Antimicrob Chemother* **68**, 471–473 (2013).
11. Caryl, J. A. & O'Neill, A. J. Complete nucleotide sequence of pGO1, the prototype conjugative plasmid from the Staphylococci. *Plasmid* **62**, 35–38 (2009).
12. Yui Eto, K. *et al*. Evolution of a 72-Kilobase Cointegrant, Conjugative Multiresistance Plasmid in Community-Associated Methicillin-Resistant Staphylococcus aureus Isolates from the Early 1990s. *Antimicrob Agents Chemother* **63**:10.1128 (2019).
